# The Crossover Effects of Supervisors’ Workaholism on Subordinates’ Turnover Intention: The Mediating Role of Two Types of Job Demands and Emotional Exhaustion

**DOI:** 10.3390/ijerph17217742

**Published:** 2020-10-23

**Authors:** Nanhee Kim, Yun Jin Kang, Jinsoo Choi, Young Woo Sohn

**Affiliations:** Psychology Department, Yonsei University, 50 Yonsei-ro, Seodaemun-gu, Seoul 06695, Korea; sksgml7025@yonsei.ac.kr (N.K.); daniropia@gmail.com (Y.J.K.); harveychoi93@yonsei.ac.kr (J.C.)

**Keywords:** workaholism, job demand, perceived workload, interpersonal conflict, emotional exhaustion, turnover intention

## Abstract

Although much research has been conducted on workaholism, its crossover effects remain uninvestigated, especially in the context of organizations. Based on the job demands-resources (JD-R) model of burnout and the conservation of resources (COR) theory, we established a dual-path structural model to examine the effects of supervisors’ workaholism on subordinates’ turnover intention through two types of job demands (perceived workload and interpersonal conflict) as well as subordinates’ emotional exhaustion. The results revealed that supervisors’ workaholism is positively related to subordinates’ emotional exhaustion through increased perceived workload and interpersonal conflict, which result in subordinates’ turnover intention. This study has made a contribution to the literature by extending the scope of workaholism research from self-perspective to other-perspective. The findings also have practical implications for organizations and their human resources (HR) practitioners.

## 1. Introduction

Since Oates [1] first defined the term workaholism as an uncontrollable need or compulsion to work, a number of studies have examined the negative consequences of the concept [2,3,4] and predictors thereof [5,6]. Although these studies have contributed meaningfully to the literature, most have focused on the domain of self-perspective. Other perspectives such as the effects of supervisors’ workaholism on co-workers and subordinates have not been explored. A few studies have investigated the impact of workaholism in the work-family area [7,8], but how workaholics affect those who work together in an organization has yet to be explored.

This study aimed to investigate how supervisors’ workaholism influences subordinates’ work attitudes. We anticipated that supervisors’ workaholism would be positively related to subordinates’ turnover intention through their emotional exhaustion. By employing the job demands-resources (JD-R) model of burnout [9] and the conservation of resources (COR) theory [10], we hypothesized that supervisors’ workaholism would have a positive effect on subordinates’ emotional exhaustion through two types of increased job demands, namely, perceived workload and interpersonal conflict, which subsequently increase their turnover intention. Research has found that job demands predict individuals’ turnover behavior and further revealed that the relationship between job demands and turnover is mediated by emotional exhaustion [11,12,13]. Consequently, the structural equational model that demonstrates the effects of supervisors’ workaholism on subordinates’ turnover intention through emotional exhaustion was established in this study. Specifically, we examined the dual mediation of perceived workload and interpersonal conflicts to explain the relationship between supervisors’ workaholism and subordinates’ emotional exhaustion.

We expected that our study would contribute to the literature, in that we extended the scope of workaholism research from self-perspective to other-perspective. We investigated the effects of supervisors’ workaholism on subordinates and the underlying mechanism of these relationships based on the JD-R model of burnout and COR theory. In addition, our study has practical implications for corporate practitioners on how to deal with supervisors’ workaholism.

### 1.1. Supervisors’ Workaholism and Subordinates’ Emotional Exhaustion

Since the term workaholism was first coined by Oates [1], although a number of studies have been conducted on the concept, there is no clear agreement on the concept except that it is a multidimensional construct. For instance, Spence and Robbins [14] defined workaholism in relation to a workaholic triad: work involvement, driveness, and enjoyment of work. However, Scott, Moore, and Miceli [15] classified workaholic behavior patterns as compulsive-dependent, perfectionist, and achievement oriented. Meanwhile, Ng and colleagues [16] defined workaholism by employing affective, cognitive, and behavioral dimensions: “those who enjoy the act of working, who are obsessed with working, and who devote long hours and personal time to work.”

Despite varying definitions and sub-dimensions of workaholism, most definitions of the concept reflect two core characteristics: working excessively (WE) and working compulsively (WC). Therefore, in this study, we employed the following definition of workaholism: “the tendency to work excessively hard in a compulsive way” [17]. WE is classified as a behavioral dimension of workaholism. Those with high workaholic tendencies are more likely to work beyond the requirements of the organization [15] and may even sacrifice their personal time [16]. WC, on the other hand, which is a cognitive dimension of workaholism, reflects workaholics’ obsession to work because of the internal pressure they place on themselves. Workaholics constantly think about their tasks even after work [18] and they experience guilt and discomfort when they are not working [14].

Most studies have examined the causes and consequences of workaholism. Research has revealed a number of predictors of workaholism such as perfectionism [6,19], self-efficacy [20,21], narcissism [22,23], conscientiousness [24,25], and overwork climate [26]. Research has also found that workaholism is detrimental to both individuals and organizations. Workaholics tend to experience low psychological wellbeing [3], low job and life satisfaction [2,15], exhaustion [4], counterproductive work behavior (CWB) [27], and low job performance [28].

Even though research on antecedents and outcomes of workaholism has contributed significantly to the literature, most studies have focused primarily on self-perspective. Although many scholars have posited that crossover effects of workaholism exist [16,29], studies either proposing a theoretical framework or empirically testing other-perspective are scarce. Moreover, workaholism studies on other-perspective have been limited to family areas, until now; for instance, research in the work-family domain has demonstrated that workaholics have a negative effect on their family members through a spillover-crossover process [30]. Since workaholics are so absorbed in their work, they are unable to execute their role in their family properly and tend to transfer their work difficulties and stress to their family. Consequently, their partners’ relational satisfaction decreases [7] and their marital estrangement increases [8]. Similarly, children of parents with workaholism have exhibited a high level of depression [31] as well as emotional and behavioral problems [32].

As such, we expected that there would be crossover effects of workaholism in the workplace as well. The impact of workaholism on others can be explained in the context of their working environment. Because workaholics are expected to have a direct and/or indirect influence on those who work in the same group [29,33], working environments that are formed through interactions between organizational members may impact workers’ attitudes and behaviors through work-related and relational factors. For example, it has been shown that supervisors’ high demands on their subordinates are positively related to subordinates’ turnover intention through increased psychological job strain [34]. In addition, Shaukat and colleagues [35] revealed that individuals feel emotionally exhausted and, consequently, want to change their jobs when there are relational conflicts among organizational members. Moreover, workaholics are likely to cause negative organizational outcomes, because they often display negative behaviors, such as incivility [36] and unrealistic performance expectations [37].

Meta-analysis research has shown that those in managerial positions are more likely to develop workaholic tendencies than other employees [27]. Since managers play an important role in organizing the working environment [38], their tendencies may be reflected in the workplace; this implies that their workaholic behavior may have an impact on the working environment. Similarly, Clark and colleagues [33] have theoretically suggested that supervisors’ workaholism may affect their subordinates’ wellbeing through the crossover process. Crossover effects can occur through two pathways. First, workaholic leaders’ negative wellbeing caused by their workaholic characteristics can influence the wellbeing of subordinates. Second, workaholic leaders’ affective, cognitive, and behavioral aspects may have a negative impact on their subordinates’ wellbeing. Specifically, leaders’ negative emotions may influence their subordinates’ feelings through emotional contagion (affect). Moreover, leaders’ workaholic behavior may make their subordinates cognitively engaged in work. This is because work–life intrusion can occur due to the characteristics of workaholics, such as continuously thinking about work (cognition). Workaholic leaders work excessively, and thus, their subordinates may also be required to work long hours as well because of an implicit norm (behavior) [33]. Further, subordinates’ excessive work tendencies caused by supervisors’ workaholism can be explained by vicarious learning [16,39]. Subordinates tend to mimic the words, attitudes, and behaviors of their workaholic supervisors because, for them, supervisors’ behaviors are considered a legitimate source of appropriate behavior in the organization [40,41]. The theoretical perspective of Clark and colleagues [33] based on affect, cognition, and behaviors can be supported by previous research. For example, it was revealed that transmission of leaders’ negative affect leads to followers’ burnout [42]. Furthermore, when people are unable to psychologically detach themselves from work during non-work hours, they tend to experience high levels of emotional exhaustion [43]. Lastly, negative organizational conditions, such as a high workload, are positively related to subordinates’ feelings of emotional exhaustion [44]. Accordingly, we expected supervisors’ workaholism to have an impact on the emotional exhaustion of their subordinates.

In accordance with the JD-R model of burnout [9], we examined the effects of supervisors’ workaholism on subordinates’ emotional exhaustion and its underlying mechanisms. The JD-R model is a theoretical framework, which attempts to explain individuals’ behaviors and psychological outcomes based on job demands and job resources. Job demands refer to “physical, psychological, social, or organizational aspects of the job that require sustained physical and/or psychological (cognitive and emotional) effort or skills” ([45], p. 312). On the contrary, job resources are defined as “physical, psychological, social, or organizational aspects of the job that are functional in achieving work goals, reducing job demands and the associated physiological and psychological costs, or stimulating personal growth, learning, and development” ([9], p. 501). Both of them have an effect on individuals’ attitudes and behaviors through strain and motivational processes, respectively. Because job demands are a key predictor of exhaustion [9] and strain process is more related to workaholism than motivational process, we examined supervisors’ workaholism by exploring two types of job demands: work-related demands and relation-related demands. Accordingly, the purpose of this study was to examine the effects of supervisors’ workaholism on subordinates’ emotional exhaustion by establishing the dual-mediation model of perceived workload and interpersonal conflict.

### 1.2. The Mediating Role of Perceived Workload

Workload is a concept that reflects the amount and difficulties of work [46,47]. When employees have a high workload, this implies that they experience a lot of pressure fulfilling their tasks [48]. In particular, subjective workload indicates the perception of having too much work to perform effectively in a given time [49], which is commonly interchangeable with work pressure and time pressure [50,51].

We assumed that supervisors’ attitudes and behaviors would have an influence on subordinates because they are involved in the same project and are in a position of authority [33,38]. Individuals with workaholism are meticulous and tend to work beyond organizational requirements [15]. They also tend to set high standards since they are likely to exhibit perfectionism [6,26,52]. Accordingly, many studies have revealed the crossover effects of supervisors’ attitudes and behaviors on their subordinates. For instance, Zhang and Seo [53] found a significant and positive relationship between supervisors’ working hours and subordinates’ working hours. Furthermore, workaholic managers with stringent standards have displayed unreasonable performance expectations of their subordinates [37]. While workaholics tend to devote a great deal of time and effort to work, they often work inefficiently [54]. To be specific, they at times spend too much time on simple tasks [55] and experience difficulties handling situations flexibly [56,57]. In this regard, we assumed that subordinates who work under supervisors with workaholism perceive a relatively high workload compared to those who work under non-workaholic supervisors.

Workload has been considered to be a key antecedent of emotional exhaustion in various occupations including office workers, airport security officers, and ministers [43,58,59,60]. The JD-R model of burnout proposes that when individuals constantly feel overloaded with work, they suffer exhaustion in the long term [9]. Workaholism is a stable individual characteristic [61], which implies that supervisors with workaholism are more likely to overtax their subordinates consistently. Moreover, their subordinates tend to experience high workloads either due to supervisors’ unreasonable expectations or inefficiency. Therefore, it is highly likely that such subordinates will perceive their supervisors’ directions as unnecessary rather than something they must do to learn and achieve, which corresponds to hindrance job demands. Hindrance job demands indicate unnecessary demands that thwart personal growth and goal attainment [62,63]. An empirical study revealed that when individuals perceive job demand as a hindrance, they show a higher level of burnout [64]. Thus, we expected subordinates to suffer from emotional exhaustion due to unnecessarily high workloads caused by their workaholic supervisors. Taken together, we posited that perceived workload would mediate the relationship between supervisors’ workaholism and subordinates’ emotional exhaustion.

### 1.3. The Mediating Role of Interpersonal Conflict

Interpersonal conflict refers to “an incompatibility between two or more opinions, principles, or interests, which leads to tension between individuals” ([65], p. 327). Interpersonal conflicts in the workplace range from trivial disagreement to ridicule and physical harassment [47]. Scholars have implicitly assumed that workaholics experience interpersonal conflicts [16,54,66]. Those with workaholism usually spend most of their time at work and, thus, think others do not work hard by comparing themselves to others [67] and tend to distrust others’ performance [56,66]. Similarly, workaholics are likely to be critical and contemptuous toward their co-workers [67] and even be hostile toward them [1,15]. In addition, while they do not trust others’ work ability, they at times complicate simple tasks [68] and act indecisively [69]. Consequently, it is plausible to assume that they will cause conflict with their subordinates in relation to their tasks.

Supervisors with high levels of workaholism are also expected to have trouble in their relationships with their subordinates because they tend to neglect relational aspects of work [54,70] and display unpleasant emotions and behaviors in front of their subordinates. Workaholism is associated with negative affect (NA) [6], and furthermore, workaholics tend to express negative emotions such as anxiety and anger to others [14,16]. Lanzo and colleagues [36] revealed a significant relationship between workaholism and incivility, which was related to aggressive workplace behavior [71]. Therefore, we assumed that those with workaholic supervisors experience interpersonal conflict with their supervisors frequently.

Scholars have long regarded interpersonal conflict as a stressful job demand [45]. Given that subordinates’ interpersonal conflicts with workaholic supervisors may be caused by disregard and rudeness shown to them, these conflicts are consumptive rather than constructive. Interpersonal conflicts at work are associated with negative emotions such as anxiety and discontent [47,72]. Even though subordinates perceive their supervisors negatively when experiencing interpersonal conflicts with them [73], they cannot express such feelings and thoughts directly because of the vertical relationship in the organization. Rather, they experience emotional labor [74,75]. Consequently, individuals are more likely to feel depleted and experience emotional exhaustion since they use their resources to control negative emotions triggered by interpersonal conflicts [76]. Research has shown that conflicts with organizational members are directly related to emotional exhaustion [77]. In particular, conflicts and unpleasant contact with supervisors lead to subordinates’ high levels of burnout [78,79]. In this regard, we posited that interpersonal conflict can mediate the relationship between supervisors’ workaholism and subordinates’ emotional exhaustion.

### 1.4. Emotional Exhaustion to Turnover Intention

Emotional exhaustion, which refers to “a feeling of being emotionally overextended and exhausted by one’s work” ([80], p. 101), is considered a core dimension of burnout [9]. Furthermore, it is associated with various negative organizational outcomes such as turnover intention [81,82] and sickness absence [83]. According to the COR theory [10], which attempts to explain individuals’ attitudes and behaviors based on their needs to preserve their resources, individuals strive to maintain and protect their resources. Therefore, where the threat of resources persists, individuals make efforts to minimize such threats. For instance, workers were found to exhibit turnover behavior in order to protect themselves from further resource loss [84,85]. In particular, individuals who are emotionally drained have a tendency to employ avoidance or withdrawal coping strategies [86]. In accordance with the COR theory, empirical studies have consistently revealed a significant positive correlation between emotional exhaustion and turnover intention [84,87], which is a well-known key predictor of turnover intention in comparison to the other dimensions of burnout–cynicism and depersonalization [88]. Thus, it was hypothesized that emotional exhaustion has a mediating effect on the relationship between supervisors’ workaholism and subordinates’ turnover intention.

### 1.5. Model

**Figure 1:**
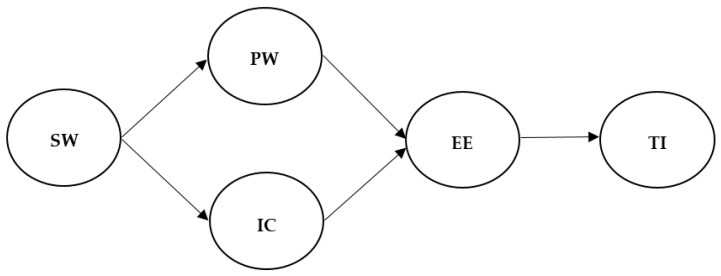
Hypothesized model. Note: SW = supervisors’ workaholism; PW = perceived workload; IC = interpersonal conflict; EE = subordinates’ emotional exhaustion; TI = subordinates’ turnover intention.

The purpose of this study was to investigate the effects of supervisors’ workaholism on subordinates’ turnover intention. Specifically, we attempted to examine the underlying mechanisms by establishing a full dual mediation model (Figure 1). Through the dual mediation of perceived workload and interpersonal conflict, supervisors’ workaholism would be positively related to subordinates’ emotional exhaustion. In turn, increased emotional exhaustion would lead to subordinates’ turnover intention. Therefore, we proposed the following hypotheses:

**Hypothesis** **1.** *Perceived workload mediates the relationship between supervisors’ workaholism and subordinates’ emotional exhaustion.*

**Hypothesis** **2.** *Interpersonal conflict mediates the relationship between supervisors’ workaholism and subordinates’ emotional exhaustion.*

**Hypothesis** **3.** *Emotional exhaustion is positively correlated with turnover intention.*

**Hypothesis** **4.** *The relationship between supervisors’ workaholism and subordinates’ turnover intention is fully mediated by perceived workload and emotional exhaustion.*

**Hypothesis** **5.** *The relationship between supervisors’ workaholism and subordinates’ turnover intention is fully mediated by interpersonal conflict and emotional exhaustion.*

## 2. Methods

### 2.1. Participants and Procedure

This study was approved by the university’s Institutional Review Board. The participants included employees from various organizations in South Korea. They were recruited through a research company that employed an online survey. The participants were asked to complete a 10-min online questionnaire for which they received a monetary reward. Employees who were not expected to work or interact frequently with their immediate supervisors such as those who were self-employed and telecommuters were excluded at the beginning of the survey.

Of the 300 participants, 56.3% were female. The average age of the participants was 35.52 years (*SD* = 8.84), ranging from 22 to 60 years. All participants had worked at their present place of employment for more than one year and their average tenure was 5.74 years (*SD* = 5.75). While the majority of the participants worked in a clerical job (68.3%), 8.7% were involved in service, 6.0% in manufacturing, 4.7% in research, 4.0% in administration, 2.3% in sales, 6.0% in others such as healthcare and education. In particular, the service sector showed the highest percentage of females (76.9%), followed by clerical job (60.5%), administration (41.7%), research (35.7%), sales (28.6%), manufacturing (11.1%), and others (61.1%). In addition, the average age distribution for each work category was as follows: manufacturing (*M* = 38.72, *SD* = 11.76), clerical job (*M* = 35.38, *SD* = 8.30), sales (*M* = 36.71, *SD* = 10.61), administration (*M* = 44.25, *SD* = 9.08), service (*M* = 34.04, *SD* = 8.99), research (*M* = 37.14, *SD* = 9.42), and others (*M* = 28.44, *SD* = 2.18).

### 2.2. Measurements

#### 2.2.1. Workaholism

We adapted The Dutch Work Addiction Scale (DUWAS) developed by Schaufeli and colleagues [89] to measure supervisors’ workaholism. Since subordinates assessed their supervisors’ workaholism level in this study, we changed “I” to “My supervisor” in the items. For example, the item “I seem to be in a hurry and racing against the clock “was changed to “My supervisor seems to be in a hurry and racing against the clock“. The scale comprises two subscales: working excessively (e.g., “My supervisor stays busy and keeps his/her irons in the fire”) and working compulsively (e.g., “I often feel that there’s something inside my supervisor that drives him/her to work hard”). Each subscale consists of five items, and a five-point Likert-type scale ranging from 1 (strongly disagree) to 5 (strongly agree) was used. To examine the psychometric properties of the modified scale, we performed confirmatory factor analysis (CFA). The results showed an acceptable fit of the data ($\chi^{2}$(34) = 116.550, *p* < 0.001, CFI = 0.93, TLI = 0.91, RMSEA = 0.08, and SRMR = 0.05), which means the psychometric properties of the modified scale were identified. DUWAS has shown acceptable internal consistency in both Dutch and Japanese samples [28,89]. The Cronbach’s alpha in this study was 0.77 for working excessively and 0.84 for working compulsively.

#### 2.2.2. Perceived Workload

The Quantitative Workload Inventory (QWI) developed by Spector and Jex [47] was employed to measure perceived workload. The scale consists of five items including “How often do you have to do more work than you can do well?” Responses were assessed on a five-point Likert scale, ranging from 1 (never) to 5 (very often). The Cronbach’s alpha was 0.77.

#### 2.2.3. Interpersonal Conflict

We adapted the eight-item interpersonal conflict scale developed by Jehn [90]. Because the original scale does not specify the subject of the interpersonal conflict, items were modified to measure interpersonal conflict with the participants’ supervisors. For example, the item “How much tension is there among members in your work unit?” was changed to “How much tension is there with your supervisor?”. The scale includes four items that measure task conflict (e.g., “How much conflict about the work you do is there with your supervisor”) and four items measuring relationship conflict (e.g., “How much are personality conflicts evident with your supervisor”). Responses were evaluated on a seven-point Likert-type scale, ranging from 1 (strongly disagree) to 7 (strongly agree). To examine the psychometric properties of the modified scale, CFA was conducted. The results showed an acceptable model fit ($\chi^{2}$(19) = 58.248, *p* < 0.001, CFI = 0.98, TLI = 0.97, RMSEA = 0.08, and SRMR = 0.02). The Cronbach’s alpha was 0.92 for task conflict and 0.90 for relationship conflict.

#### 2.2.4. Emotional Exhaustion

The Maslach Burnout Inventory-General Survey (MBI-GS) developed by Maslach, Schaufeli, and Leiter [91] was used to measure emotional exhaustion. Only the emotional exhaustion subscale of the MBI-GS was employed in the study. The subscale comprises five items including “I feel emotionally drained by my work” and a five-point Likert-type scale ranging from 1 (strongly disagree) to 5 (strongly agree) was used. The Cronbach’s alpha was 0.89.

#### 2.2.5. Turnover Intention

Turnover intention was measured using a three-item scale developed by Lee, Kim, and Park [92]. Two of the items are “I often think of changing my job” and “I plan to look for a new job within the next 1 year”. Responses were assessed on a five-point Likert-type scale, ranging from 1 (strongly disagree) to 5 (strongly agree). The Cronbach’s alpha was 0.86.

#### 2.2.6. Control Variables

In order to control exogenous effects, several demographic variables, namely, age, sex, job tenure, and occupational type were controlled. Demographic variables were measured in the following ways: age (in years), sex (0 = male, 1 = female), job tenure (in years), and occupational type (1 = manufacturing, 2 = clerical work, 3 = sales, 4 = administration, 5 = service, 6 = research, 7 = other). Occupational type was dummy-coded.

### 2.3. Analysis

Descriptive statistics (means, standardized deviations, correlations) and internal consistencies were analyzed by utilizing SPSS 25.0. Subsequently, based on Andersen and Gerbing’s guidelines [93], we performed CFA by using Mplus 8.0 to evaluate our measurement model. Thereafter, structural equation modeling (SEM) with 95% bias-corrected confidence intervals (5000 bootstrapped samples; [94]) was conducted to test our hypotheses. The model fit of the data was examined with $\chi^{2}$, comparative fit index (CFI), Tucker-Lewis index (TLI), the root-mean-square error of approximation (RMSEA), and standardized root-mean-square residual (SRMR). Values greater than 0.9 for both CFI and TLI and values less than 0.08 for both RMSEA and SRMR were considered acceptable [95].

## 3. Results

### 3.1. Preliminary Analysis

Before testing our model, we conducted preliminary analyses using SPSS 25.0 to check basic statistical assumptions [96]. The results revealed that there was no multicollinearity in our data. Furthermore, no univariate or multivariate outlier was found. In addition, when we tested the normality of the data, skewness and kurtosis were below the thresholds (|skewness| < 3, |kurtosis| < 5; [97]).

### 3.2. Descriptive Statistics

The means, standard deviations, and correlations of the variables are presented in Table 1. With the exception of several relationships with control variables, all the variables showed significant correlations, as expected.

### 3.3. Measurement Model

We performed CFA to test our measurement model (five-factor model). When conducting CFA and SEM, we used the scale items of each variable as observed indicators for constructing latent variables of perceived workload, emotional exhaustion, and turnover intention. On the contrary, following Weston and Gore’s recommendation [96], we used sub-dimensions as observed indicators for constructing latent variables of supervisors’ workaholism and interpersonal conflict. The results showed an acceptable fit of the data, $\chi^{2}$(109) = 255.261, *p* < 0.001, CFI = 0.94, TLI = 0.93, RMSEA = 0.07, and SRMR = 0.06. Furthermore, our measurement model demonstrated better fit indices than other measurement models, combining all the variables into one latent factor ($\chi^{2}$(119) = 1131.924, *p* < 0.001, CFI = 0.61, TLI = 0.55, RMSEA = 0.17, and SRMR = 0.11).

### 3.4. Structural Model

We conducted our structural model testing after controlling for age, sex, tenure, and occupational type. Although we first hypothesized the full mediation model, we began our analyses with a partial mediation model including all feasible paths in order to leave other possibilities open. The results indicated a good fit ($\chi^{2}$(161) = 325.710, *p* < 0.001, CFI = 0.94, TLI = 0.92, RMSEA = 0.06, and SRMR = 0.06). However, no significant direct paths were found with the exceptions of those we assumed. The results revealed that supervisors’ workaholism was not significantly associated with subordinates’ emotional exhaustion (β = −0.01, *p* = 0.93) and turnover intention (β = 0.05, *p* = 0.42). Likewise, both perceived workload and interpersonal conflict did not show any relationship with turnover intention: perceived workload (β = −0.01, *p* = 0.88) and interpersonal conflict (β = 0.11, *p* = 0.11).

Thereafter, we analyzed our full mediation model after removing non-significant paths from the partial mediation model. Likewise, it also showed a good model fit, $\chi^{2}$(165) = 330.463, *p* < 0.001, CFI = 0.94, TLI = 0.92, RMSEA = 0.06, and SRMR = 0.06. Thus, we continued our hypothesis testing using the full mediation model, which is more parsimonious.

**Figure 2:**
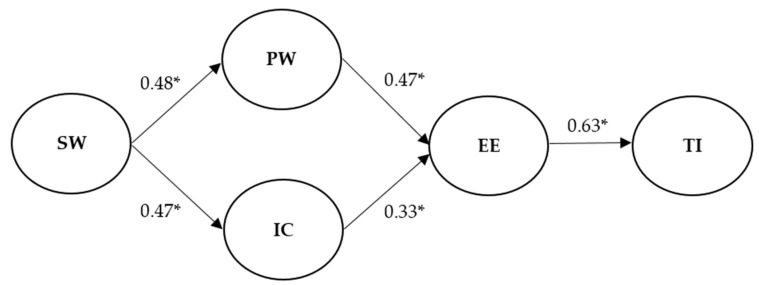
Structural model with standardized path estimates. Note: SW = supervisors’ workaholism; PW = perceived workload; IC = interpersonal conflict; EE = subordinates’ emotional exhaustion; TI = subordinates’ turnover intention; * *p* < 0.001.

As depicted in Figure 2, supervisors’ workaholism showed a positive association with perceived workload (β = 0.48, *p* < 0.001) and interpersonal conflict (β = 0.47, *p* < 0.001). In addition, both perceived workload (β = 0.47, *p* < 0.001) and interpersonal conflict (β = 0.33, *p* < 0.001) were positively related to emotional exhaustion. In addition, supervisors’ workaholism and subordinates’ emotional exhaustion were indirectly related through perceived workload (β = 0.23, *p* < 0.001, bootstrap 5000 samples, 95% CI [0.14, 0.32]) and interpersonal conflict (β = 0.15, *p* < 0.001, bootstrap 5000 samples, 95% CI [0.08, 0.23]). Thus, Hypothesis 1 and Hypothesis 2 were supported. Furthermore, there was a positive relationship between emotional exhaustion and turnover intention (β = 0.63, *p* < 0.001), thus supporting Hypothesis 3. Lastly, supervisors’ workaholism had a significant indirect effect on subordinates’ turnover intention through perceived workload and emotional exhaustion (β = 0.14, *p* < 0.001, bootstrap 5000 samples, 95% CI [0.09, 0.20]) as well as through interpersonal conflict and emotional exhaustion (β = 0.10, *p* < 0.001, bootstrap 5000 samples, 95% CI [0.05, 0.15]). The results of indirect effects are presented in Table 2. Therefore, Hypothesis 4 and Hypothesis 5 were also supported. In essence, all the hypotheses were supported.

## 4. Discussion

Our study tried to investigate the effects of supervisors’ workaholism on subordinates’ turnover intention. As we hypothesized, the results revealed that supervisors’ workaholic tendencies are positively related to subordinates’ emotional exhaustion through increased perceived workload and interpersonal conflict. Moreover, subordinates’ increased emotional exhaustion consequently leads to turnover intention. This study contributes to the literature in that it has extended the scope of workaholism research from self-perspective to other-perspective. While previous studies have focused on the domain of self-perspective, we shed light on the effects of leaders’ workaholism on others, specifically subordinates. Although some studies have suggested the possible effects of leaders’ workaholic behaviors on subordinates’ attitudes and behaviors, not much empirical research has been conducted on this. For instance, it has been implicitly assumed that workaholics will have trouble with others because they are only absorbed in work and, thus, neglect their relationships with others [16,54]. In line with this, our study empirically showed a positive relationship between supervisors’ workaholism and interpersonal conflict. Moreover, according to Clark et al. [33], supervisors’ long working hours may be transferred to subordinates because of implicit norms, and furthermore, they may suffer increased stress due to task-intrusion caused by their workaholic supervisors. These assumptions are consistent with our results in that supervisors’ workaholism leads to subordinates’ emotional exhaustion due to their perceptions of an increased workload. Therefore, this study contributes to the literature in that it lays the groundwork for further workaholism research based on other-perspective by empirically investigating implicit assumptions of workaholism.

In addition, this study has contributed theoretically by identifying psychological mechanisms of the positive effects of supervisors’ workaholism on subordinates’ turnover intention based on the JD-R model of burnout [9] and the COR theory [10]. Our results revealed that the relationship between supervisors’ workaholism and subordinates’ turnover intention was fully mediated by two types of job demands, namely, perceived workload and interpersonal conflict as well as emotional exhaustion. Therefore, we established full mediation based on the JD-R model of burnout and COR theory. Although our study revealed a full mediation process between supervisors’ workaholism and subordinates’ turnover intention, the results need to be interpreted carefully. In particular, the results did not indicate a direct path between interpersonal conflict and turnover intention. However, because several previous studies have reported a direct relationship between interpersonal conflict and turnover intention [98,99], further research using various samples will be required to verify our results so that generalizations can be made.

Furthermore, with the exception of perceived workload and interpersonal conflict, additional research on possible mediators is recommended. According to the JD-R model, various working conditions can be classified into two categories, namely, job demands and job resources. Demerouti et al. [9] asserted that job resources influence task performance and can lead to withdrawal behavior. In this regard, we can expect that workaholic supervisors’ effects on their subordinates may be caused by not only increased job demands but also a lack of job resources. Accordingly, additional research could be conducted to explore the effects of supervisors’ workaholism on their subordinates from the perspective of job resources.

In a similar vein, it is recommended that further research on possible moderators of workaholism be conducted. The JD-R model [9] posits that there are interaction effects between job demands and job resources. Furthermore, it has been suggested consistently that job resources function as a moderator, which mitigates psychological and physical costs derived from job demands [40]. For example, studies have revealed that job resources such as autonomy and social support can buffer the impact of workload on an individual’s exhaustion [100,101]. In accordance with these findings, we expected that the negative influence of supervisors’ workaholism could be moderated by the extent of supervisors or organizations’ provision of job resources to their subordinates.

Besides theoretical implications, our study also has practical implications for employers and HR practitioners. Employees’ turnover behavior not only results in short-term organizational costs and losses but also affects other members’ turnover in the long term, thus reducing organizational effectiveness [102]. Our findings imply that organizations can implement interventions in order to prevent employees’ turnover. For instance, enhancing role clarity may moderate the negative effects of supervisors’ workaholism. Research has demonstrated that role clarity has a negative association with burnout [88,103]. Moreover, high levels of role clarity can moderate exhaustion and psychological strain caused by work overload [104]. Thus, employees’ emotional exhaustion can be reduced by providing coaching or training programs that enable supervisors to give clear instructions to their subordinates.

In the context of the JD-R model, a negative working environment can be mitigated through changes in the organization or individuals [105]. Our results showed that increased job demands have a negative effect on individuals. However, if individuals perceive such job demands as meaningful or enhancing their personal growth, the negative effects could be reduced or eliminated [106]. For example, corporate social responsibility (CSR) is known to help employees experience meaningfulness in their work [107]. In this regard, organizations can help employees to find meaning in their work and mitigate negative experiences from high job demands by their active participation in CSR. Moreover, at an individual level, job crafting improves the meaning individuals ascribe to work by changing their tasks, relationships, and cognition [48,108]. Thus, corporate counselors and HR practitioners should encourage employees to perform such job crafting activities by providing coaching and training and conducting seminars. Together, organizations’ efforts in CSR and individuals’ job crafting behaviors may mitigate the negative role of supervisors’ workaholism.

### Limitations and Recommendations for Future Studies

Despite its theoretical and practical contributions, this study has several limitations. First, causal inference cannot be guaranteed due to the cross-sectional design employed. Although the hypothesized relationships were based on theories, causal relationships cannot be determined through cross-sectional data. Consequently, a further longitudinal study is recommended to determine causality. Second, subordinates assessed their supervisors’ workaholism. We assumed that rating of their supervisors’ workaholism would not be problematic because the previous study has shown that self-rating and coworker-rating of workaholism were not significantly different [109]. In addition, when we performed CFA for the modified scale, our data was well fitted to a two-factor model, the same as the original scale. However, Mazzetti and colleagues [110] recently suggested that the ratings of WC can be varied depending on the source of response (e.g., self, observer). In their study, WE and the sum score of workaholism were not significantly different depending on the rater, except WC. We assumed that such results were due to the characteristics of WC, which measures individuals’ cognition. Therefore, future research is needed on whether the rating of WC is actually different, depending on the source of the response. In addition, further information acquired through various response sources might provide valuable information. For instance, a study showed that a gap between leaders and followers’ perceptions for transformation leadership is related to negative organizational culture, particularly when leaders rate themselves more positively [111]. Accordingly, we can postulate that the negative effects of workaholism will be greater when subordinates rate their supervisors as highly workaholic while the supervisors do not. Third, our study explored the effects of supervisors’ workaholism by focusing on the job demand dimension in the JD-R model. It is recommended that studies based on job resources be conducted to identify how interactions between job demands and job resources affect subordinates’ emotional exhaustion and turnover intention. Finally, the sample of this study was limited to South Korean workers. In comparison to western countries, individuals from eastern countries are more vulnerable to others’ influence due to their notions of collectivism and hierarchical characteristics [112]. Although studies have consistently identified the negative effects of workaholism regardless of the country [89,113], our results may have been due to cultural characteristics. Thus, further research based on western samples will be required to generalize the results of our study.

## 5. Conclusions

We have attempted to broaden the literature on workaholism by examining the effects of supervisors’ workaholism on subordinates’ work attitudes and behaviors. Our results revealed that supervisors’ workaholism affects subordinates’ turnover intention through their increased emotional exhaustion. Furthermore, by utilizing the JD-R model of burnout and COR theory, the underlying mechanisms of its indirect effects were explored in this study. Our results revealed that both perceived workload and interpersonal conflict fully mediate the association between supervisors’ workaholism and subordinates’ emotional exhaustion. In turn, increased emotional exhaustion leads to turnover intention. This study contributes to the literature by extending workaholism research from the self- to other-perspective. Furthermore, implicit assumptions of the effects of workaholism on others were investigated empirically. This study also has several practical implications. HR managers could reduce employees’ turnover intention through organizational interventions such as coaching and counseling programs on leadership and job crafting. Finally, future research based on diverse samples that employ longitudinal designs are required to generalize the results.

## Figures and Tables

**Table 1:** Descriptive statistics.

Variables	*M*	*SD*	1	2	3	4	5	6
1. Age	35.52	8.84	-					
2. Tenure	5.74	5.75	0.64 **	-				
3. Supervisors’ Workaholism	2.86	0.72	0.07	−0.04	-			
4. Perceived Workload	3.20	0.67	−0.01	−0.02	0.41 **	-		
5. Interpersonal Conflict	3.39	1.32	0.11 *	0.09	0.39 **	0.33 **	-	
6. Emotional Exhaustion	3.24	0.86	−0.09	−0.04	0.30 **	0.48 **	0.44 **	-
7. Turnover Intention	2.95	1.07	−0.22 **	−0.22 **	0.20 **	0.30 **	0.32 **	0.55 **

Note: *N* = 300; * *p* < 0.05, ** *p* < 0.01.

**Table 2:** Indirect relations for the structural model.

Paths	Standardized Indirect Effect		Bootstrap Bias Corrected 95% Confidence Interval	
	*β*	*SE*	*Lower Bound*	*Upper Bound*
SW → PW → EE	0.23	0.04	0.14	0.32
SW → IC → EE	0.15	0.03	0.08	0.23
SW → PW → EE → TI	0.14	0.03	0.09	0.20
SW → IC → EE → TI	0.10	0.02	0.05	0.15

Note: SW = supervisors’ workaholism; PW = perceived workload; IC = interpersonal conflict; EE = subordinates’ emotional exhaustion; TI = subordinates’ turnover intention.
